# The Diuretic Effects of Coconut Water by Suppressing Aquaporin and Renin–Angiotensin–Aldosterone System in Saline-Loaded Rats

**DOI:** 10.3389/fnut.2022.930506

**Published:** 2022-06-23

**Authors:** Jing Wei, Mantong Zhao, Keke Meng, Guanghua Xia, Yonggui Pan, Congfa Li, Weimin Zhang

**Affiliations:** ^1^Hainan Engineering Research Center of Aquatic Resources Efficient Utilization in South China Sea, Key Laboratory of Food Nutrition and Functional Food of Hainan Province, Key Laboratory of Seafood Processing of Haikou, College of Food Science and Engineering, Hainan University, Haikou, China; ^2^Key Laboratory of Tropical Fruits and Vegetables Quality and Safety for State Market Regulation, Hainan Institute for Food Control, Haikou, China

**Keywords:** coconut water, coconut (*Cocos nucifera* L.), water-electrolyte metabolism, polyphenols, AQP

## Abstract

The acute and prolonged diuretic effects of coconut water (CW) and the underlying mechanism were investigated with a saline-loaded rat model. In an acute diuretic experiment, CW could significantly increase urine excretion. In addition, the treatment of CW significantly increased urinary sodium and chloride ions, thereby considerably increasing the excretion of NaCl. However, the calcium concentration and pH value were not affected. In the prolonged diuretic experiment, CW dramatically increased the urine output and urine electrolyte concentrations (Na^+^, K^+^, and Cl^–^). Furthermore, CW could suppress the activation of renin–angiotensin–aldosterone system by decreasing serum antidiuretic hormone, angiotensin II, and aldosterone levels, and significantly increasing the serum atriopeptin level. CW treatment significantly reduced the mRNA expressions and protein levels of aquaporin 1 (AQP1), AQP2, and AQP 3. This report provided basic data for explaining the natural tropical beverage of CW as an alternative diuretic agent.

## Introduction

Diuretics like furosemide and thiazides have determinately shown their efficiency in treating edema, hypertension, cardiovascular diseases, and other metabolic diseases (1–3). However, these drugs usually exhibit several adverse effects, such as electrolyte disturbances, metabolic disorders, and acute hypovolemia after long periods of intake (4, 5). Therefore, it is meaningful to seek safe and effective diuretic alternatives from natural products. In recent years, some phytochemicals extracted from medicinal plants such as *Tropaeolum majus*, *Fumaria species*, *Lagopsis supina*, and *Herniaria glabra L*., were reported to exhibit excellent diuretic activities with little undesired adverse effect, which opened the sight for the exploitation of natural and safe diuretics (6–10).

Coconut water is an ancient tropical beverage containing substantial minerals, vitamins, sugars, amino acids, and polyphenol compounds, which is favored by consumers due to its nutritional characteristics and distinctive flavor (11, 12). Coconut water not only exerts obvious antioxidant activity but also exhibits various pharmacological activities like anti-inflammatory, antioxidant, antithrombotic, and diuresis renal regenerative actions, which play vital roles in health promotion and medicinal application, which could also be injected intravenously in an emergency (11–13). When used for hypertension treatment, coconut water could significantly decrease the blood pressure and cause frequent urination, which indicates its potential diuretic effects without electrolyte disorder. Furthermore, the potassium level in the plasma was increased after consuming coconut water termly, which could serve as a crucial factor in the anti-hypertension ability, indicating a potassium-sparing natriuretic (12, 13).

At present, little study has already been done previously to determine the diuretic effect of coconut water, and the underlying mechanism remains unclear. In some previous studies, the glycosides and flavonoids extracted from plant *Lagopsis supina* were observed to exhibit a significant diuretic effect via suppression of aquaporins (AQPs) and renin–angiotensin–aldosterone system (RAAS) pathways in saline-loaded rats (9). Therefore, the current study aimed to investigate the acute and prolonged diuretic effect of coconut water and underlying mechanisms in the saline-loaded rat model after characterization of phytochemical constituents in coconut water by ultrahigh-performance liquid chromatography-quadrupole time-of-flight tandem mass spectrometry (UPLC-Q-TOF-MS/MS). The urine excretion volume, pH values, and urinary electrolyte concentrations were determined to evaluate its diuretic activity. Meanwhile, the Na^+^–K^+^–ATPase, antidiuretic hormone (ADH), aldosterone (ALD), angiotensin II (Ang II), atriopeptin (ANP) levels, mRNA, and the expressions of AQP1, AQP2, and AQP3 in serum were also determined to reveal the potential mechanism as well.

## Materials and Methods

### Chemicals and Reagents

Fresh golden coconuts (*Cocos nucifera* L.cv.Wenye.No. 4) were obtained from a local market (Haikou, Hainan, China). LC-MS grade acetonitrile was purchased from Spectrum Chemical Mfg. Corp. (Gardena, CA, United States). Deionized water was purified by a Milli-Q water system (Merck Millipore, Milford, CT, United States). Pentobarbital sodium was purchased from Hainan Qiyuan Biological Technology Co., Ltd. (Haikou, China). All other agents of the analytical reagent grade were purchased from Kemiou Chemical Reagent Co., Ltd. (Tianjin, China). Furosemide (a reference loop diuretic drug) was obtained from Hainan Qilu Pharmaceutical Co., Ltd. (Haikou, China).

### Coconut Water Preparation

The fresh coconuts were washed with deionized water and then cut for fresh coconut water sampling. After vacuum filtration, the clarified coconut water was collected and stored at −20°C in the refrigerator until use.

### Determination of Polyphenols Compounds Composition and Amino Acid Composition of Coconut Water

The phytochemical profiles of coconut water, especially for the polyphenols compounds composition, were determined by using UPLC–Q-TOF–MS/MS including Shimadzu UHPLC System (Kyoto, Japan) tandem with an AB SCIEX 5600 system (Foster City, CA, United States) according to a previous study (9).

### Determination of Soluble Matter Proportion of Coconut Water

The coconut water was lyophilized with a Scientz-10ND vacuum freeze-dryer (Ningbo Scientz Biotechnology Co., Ltd., Zhejiang, China) and the soluble matter ratio (g/L) of coconut water was determined. The coconut water (CW-L) and the concentrated coconut water (CW-H) were restored with normal saline in the ratio of normal saline to soluble components at 1:1 and 1:2, respectively.

### Animals Experiment Design

Male Sprague–Dawley rats (Certificate no. SCXK2019-0014, 14 w, 200–250 g) were purchased from Changsha Tianqin Biological Technology Co., Ltd. (Changsha, China) and kept in a temperature and a relative humidity-controlled room (23°C, 70–80%) with 12 h dark and light cycle. All rats had free access to food and water. All animal procedures obeyed the guidelines of the care of experimental animals of China Animal Health and Epidemiology Center.

### Acute Diuretic Activity

#### Experimental Design

The experiment was performed according to Pãltinean et al. (7) with slight modification as follows: after adaptive feeding for 1 week, 40 rats were randomly divided into four groups (*n* = 10 per group) and were treated by gavage with the same volume of normal saline with dose 10 mL/kg⋅bw (CON group), 10 mL/kg⋅bw of 10 mg/kg furosemide (F-10 group), coconut water (CW-L group), and concentrated coconut water (CW-H group), respectively. Then, the rats were immediately transferred to a ventilated independent metabolic cage. The urine excretion volume was recorded at 6 and 24 h. After sacrifice by anesthesia, the blood of the rats was collected and allowed to stand at room temperature for 60 min until coagulation. Then it was left to stand for another 30 min at 4°C and centrifuged at 3,000 × *g* for 20 min to obtain the serum, which was stored at –80°C for further analysis.

#### Diuretic Effect Assay

The diuretic effect of coconut water on rats was evaluated by urinary excretion (1), diuretic index (2), and diuretic activity (3) (14):


(1) $$\mathrm{Urinary}\,\mathrm{excretion}=\frac{\text{Totalu}\,\mathrm{rinary}\,\mathrm{output}}{\mathrm{Total}\,\mathrm{liquid}\,\mathrm{administered}}$$

(2) $$\mathrm{Diuretic}\,\mathrm{index}=\frac{\mathrm{Urinary}\,\mathrm{excretion}\,\mathrm{of}\,\mathrm{treatment}\,\mathrm{groups}}{\mathrm{Urinary}\,\mathrm{excretion}\,\mathrm{of}\,\mathrm{control}\,\mathrm{group}}$$

(3) $$\mathrm{Diuretic}\,\mathrm{activity}=\frac{\mathrm{Diuretic}\,\mathrm{index}\,\mathrm{of}\,\mathrm{test}\,\mathrm{drug}}{\mathrm{Diuretic}\,\mathrm{index}\,\mathrm{of}\,\mathrm{standard}\,\mathrm{drug}}$$

#### Urinary Electrolyte Level Assay

Urinary electrolyte levels (Cl^–^, Na^+^, K^+^, and Ca^2+^ concentrations) were determined after 24 h using commercial reagent kits from Nanjing JianCheng Institute of Biotechnology Co., Ltd. (Nanjing, China) according to the appended instruction. Saliuretic index, natriuretic index, and carbonic anhydrase index (CAI) were calculated according to the following formula (15–17):


(4) $$\mathrm{Saliuretic}\,\mathrm{index}=\frac{\mathrm{Urinary}\,N\,{\text{a}}^{+},{\text{K}}^{+},{\mathrm{Cl}}^{-}\,\mathrm{level}\,\mathrm{in}\,\mathrm{the}\,\mathrm{test}\,\mathrm{group}}{\mathrm{Urinary}\,{\mathrm{Na}}^{+},{\text{K}}^{+},{\text{Cl}}^{-}\,\mathrm{level}\,\mathrm{in}\,\mathrm{the}\,\mathrm{control}\,\mathrm{group}}$$

(5) $$\mathrm{Natriuretic}\,\mathrm{index}=\frac{\mathrm{Urinary}\,{\mathrm{Na}}^{+}\,\mathrm{level}\,\mathrm{in}\,\mathrm{the}\,\mathrm{same}\,\mathrm{test}\,\mathrm{group}}{\mathrm{Urinary}\,K^{+}\,\mathrm{level}\,\mathrm{in}\,\mathrm{the}\,\mathrm{same}\,\mathrm{test}\,\mathrm{group}}$$

(6) $$\mathrm{CAI}\,\mathrm{index}=\frac{\mathrm{Urinary}\,{\mathrm{Cl}}^{-}\,\mathrm{level}\,\mathrm{in}\,\mathrm{the}\,\mathrm{same}\,\mathrm{test}\,\mathrm{group}}{\mathrm{Sum}\,\mathrm{of}\,\mathrm{urinary}\,{\mathrm{Na}}^{+}+K^{+}\,\mathrm{level}\,\mathrm{in}\,\mathrm{the}\,\mathrm{same}\,\mathrm{test}\,\mathrm{group}}$$

### Prolonged Diuretic Activity

#### Experimental Design

The experiment was performed according to Yang et al. (9) with slight modification as follows: 36 rats were given 5 mL/100 g of normal saline to apply the same level of saline load before treatment. They were randomly divided into four groups (*n* = 9 per group), which were treated by gavage with 10 mL/kg⋅bw of normal saline (CON group), 10 mL/kg⋅bw furosemide of 10 mg/kg (F-10 group), coconut water (CW-L group), and concentrated coconut water (CW-H group). Then, the rats were immediately transferred to a ventilated independent metabolic cage. The urine excretion volume was measured after 24 h. After administration of different compounds and monitoring of body weight, food consumption, and water intake once a day for consecutive 7 days, all rats were sacrificed after anesthesia with 10% chloral hydrate. The blood samples were collected and allowed to stand at room temperature for 60 min until coagulation. After standing for 30 min at 4°C it was centrifuged at 3,000 × *g* for 20 min to obtain serum and stored at –80°C for further analysis (18).

#### Urinary Electrolyte Level Assay

The 24-h urinary excretion volume of all groups was measured and calculated. The Cl^–^, Na^+^, and K^+^ concentrations in rat urines were determined using commercial reagent kits from Nanjing JianCheng Institute of Biotechnology Co., Ltd. (Nanjing, China) according to the instructions of the manufacturer.

#### Renin–Angiotensin–Aldosterone System Factors Analysis

The Na^+^–K^+^–ATPase activity and related proteins including ADH, ALD, Ang II, ANP, and membrane proteins in aquaporins (AQPs) including AQP1, AQP2, and AQP3 levels in serum were determined using commercial enzyme-linked immunosorbent assay (ELISA) kits from Shanghai MLBIO Biological Technology Co., Ltd. (Shanghai, China) based on the manufacturer’s instructions.

#### qRT-PCR and Western Blot

The mRNA expressions of genes such as AQP1, AQP2, and AQP3 that regulate kidney tissue function and urine formation were detected by RT-PCR. The procedure of RT-PCR was performed according to our previous work (19, 20). Briefly, after the kidneys were ground and homogenized, total RNA was extracted based on the kit instructions. The concentration and purity of total RNA were determined by an ultra-micro protein nucleic acid analyzer. RNA of the kidney tissue sample (2 μg) was reverse transcribed to cDNA. The qRT-PCR determination was conducted as follows: pre-denaturation 10 min at 95°C, 40 cycles of 15 s at 95°C, 30 s at 60°C, another 30 s at 72°C, and 10 min at 72°C for a final extension. The relative expression level of genes was expressed as the ratio of target gene expression level and the level of GAPDH gene expression for the data normalization. The primers of AQP1, AQP2, AQP3, and GAPDH used in this study were designed by Sangon Biotech Co., Ltd. (Shanghai, China), with the sequences described in Table 1.

**TABLE 1:** Primer used for the real-time polymerase chain reaction (qRT-PCR).

Genes	Sequence (5′-3′)	
AQP1	F-ATTGGCTTGTCTGTGGCTCTTGG	R-TGGTTTGAGAAGTTGCGGGTGAG
AQP2	F-CTTCCTTCGAGCTGCCTTCTATGTG	R-GCTGTGGCGTTGTTGTGGAGAG
AQP3	F-CTGTGGTTCCGTGGCTCAAGTG	R-GATGGCAAGGGTGACAGCGAAG
GADPH	F-TGCCACTCAGAAGACTGTGG	R-TTCAGCTCTGGGATGACCTT

The protein expressions of related genes such as AQP1, AQP2, and AQP3 were evaluated and confirmed by Western blotting based on the procedure reported in our previous work (21–23). Firstly, the total protein was separated using gel electrophoresis and transferred to a PVDF membrane that had been incubated with primary antibodies at 4°C overnight and incubated with secondary antibodies orderly. At last, the protein bands were visualized and used for analysis. The primary antibodies of AQP1 (1:10,000, Abcam), AQP2 (1:500, Abcam), AQP3 (1:1,000, Abcam), and β-actin (1:200, Abcam), and the secondary antibodies from horseradish peroxidase-conjugated (1:1,000, CST) were used in this work. The relative protein level of genes was expressed as the ratio of target gene expression level and β-action gene expression level for the data normalization.

### Statistical Analysis

Statistical analysis was conducted using SPSS 19.0 software (SPSS Inc., Chicago, IL, United States), and the data were expressed as means ± standard deviation (SD). One-way analysis of variance (ANOVA) and Dunnett’s test were used to compare the model group and treated group. Gene expression levels comparison between different groups was conducted by Mann-Whitney *U* test. *p < 0.05 and ^**^p < 0.01 compared with the model group.

## Results

### Polyphenols and Amino Acid Composition in Coconut Water

After identifying the molecular structure of polyphenols based on the molecular fragments’ information in the secondary spectrum, their contents were determined. As shown in Table 2, totally, 37 compounds, including 19 flavonoids, 7 anthocyanins, 2 benzaldehydes, and 9 phenolic acids were identified from CW. Among them, chlorogenic acid, procyanidin, L-epicatechin, cinnamic acid, sinapic acid, and baicalein were abundant. The content of total amino acids reached 588.2 mg/L. A total of 18 species of amino acids were characterized, and among them, alanine (Ala), proline (Pro), and phenylalanine were abundant (Supplementary Table 1).

**TABLE 2:** Polyphenols compounds compositions of coconut water.

Polyphenols	Count	Rt/min	Species	Content (ng/mL)
Flavones	1	10.72	Apigenin	230
	2	13.51	Apigetrin	1162
	3	11.45	Baicalein	1760
	4	7.07	Apigenin8-C-glucoside	50.3
	5	7.09	Liquiritin	120.1
Flavonols	6	8.24	Myricetin	587
	7	7.20	Isoquercitrin	70.1
	8	8.13	Guaijaverin	651
	9	8.06	Isorhamnetin-3-rutinoside	107
	10	8.24	Isorhamnetin-3-o-β-D-glucoside	80.5
	11	7.13	Rutinum	188
Flavonones	12	11.01	Naringenin	42.9
Flavanonols	13	6.82	Taxifolin	125.6
Flavan-3ols	14	5.14	Catechin	992
	15	5.13	L-Epicatechin	3900
	16	6.94	Epicatechin gallate	184
	17	4.46	Procyanidin B1	7890
	18	4.45	Procyanidin B2	6430
Isoflavones	19	10.8	Genistein	30.8
Anthocyanins	20	5.80	(+)-Leucocyanidin	143
	21	5.18	Cvanidin-3-o-glucoside	71.4
	22	0.68	Phloroglucinol	330
	23	6.83	Polydatin	76.3
	24	8.82	*trans*-3,4′,5-trihydroxistilbene	154
	25	3.23	Carvacrol	4150
	26	7.36	Sinapine	1956
Benzaldehydes	27	7.17	Ethyl vanillin	796
	28	5.04	Vanillin	396
Phenolic acids	29	3.76	Caffeic acid	440.2
	30	12.84	Sinapic acid	5715
	31	15.9	Chlorogenic acid	99100
	32	6.38	Ferulic acid	82.5
	33	5.42	p-Coumaric acid	233
	34	1.27	Protocatechuic acid	54.9
	35	3.52	Vanillic acid	188.8
	36	13.48	Cinnamic acid	4894
	37	14.70	Rosmarinic acid	336.5

### Acute Diuretic Activity of Coconut Water on Rats

#### The Effect of Acute Treatment With Coconut Water on Urine Excretion of Rats

To determine the acute diuretic effects of coconut water, the urine volume of CON, F-10, CW-L, and CW-H groups was determined after 6 and 24 h (Table 3). Compared to the CON group, the urine volume of F-10 and CW-L increased significantly at 6 and 24 h (*P* < 0.01), except F-10 at 6 h (*P* < 0.05). However, the urine volume of CW-H group did not increase significantly. In terms of diuretic activity, the diuretic activity of CW-L group was 1.19, while the CW-H was 0.82, indicating that the CW-L group had a better effect on promoting urine volume.

**TABLE 3:** The effect of acute treatment with coconut water on urinary excretion volume at 6 and 24 h in rats.

Groups	6 h				24 h			
	Volume of urine (mL/100 g)	Urinary excretion	Diuretic action	Diuretic activity	Volume of urine (mL/100 g)	Urinary excretion	Diuretic action	Diuretic activity
CON	1.72 ± 0.24	0.86 ± 0.12	–	–	4.71 ± 0.81	2.37 ± 0.48	–	–
F-10	2.01 ± 0.22	1.00 ± 0.08	1.16	–	7.41 ± 1.19**	3.73 ± 0.79**	1.58	–
CW-L	2.37 ± 0.46**	1.18 ± 0.24*	1.38	1.18	8.77 ± 1.07**	4.40 ± 0.72**	1.86	1.18
CW-H	2.09 ± 0.50	1.05 ± 0.30	1.23	1.05	6.16 ± 1.91	3.13 ± 1.17	1.32	0.84

*p < 0.05 and **p < 0.01 compared with the model group.

#### The Effect of Acute Treatment With Coconut Water on Urinary Electrolyte Concentrations

Compared to the CON group, the urine Na^+^ and Cl^–^ concentrations in the F-10 group were significantly increased after 24 h (*P* < 0.01). After acute treatment with CW, the urine Na^+^ and Cl^–^ concentrations in CW-L and CW-H groups were significantly increased (*P* < 0.01), and a higher concentration of CW enhanced the improvement, while the pH values and concentrations of Ca^2+^ were hardly affected. The ratio of Na^+^/K^+^ in the CON group was 1.69 and the intake of furosemide and CW significantly enhanced the rats’ urinary natriuretic index (Na^+^/K^+^), which were 1.85 1.87, and 1.89 in the F-10, CW-L, and CW-H groups, respectively. Compared with the CON group, the urine CAI index of CW-L and CW-H groups increased (Table 4).

**TABLE 4:** Effects of acute treatment with coconut water on the urine pH values and electrolyte concentrations of rats (24 h).

Groups	pH	Urine electrolyte (mmol/L)				Saliuretic index			Na^+^/K^+^	Cl^–^/(Na^+^/K^+^)
		Na^+^	K^+^	Cl^–^	Ca^2+^	Na^+^	K^+^	Cl^–^		
CON	9.01 ± 0.10	111.46 ± 7.77	65.88 ± 10.52	142.91 ± 10.45	1.37 ± 0.17	1.00	1.00	1.00	1.69	0.81
F-10	8.83 ± 0.23	129.89 ± 8.67**	70.18 ± 8.06	200.37 ± 22.39**	1.11 ± 0.16	1.17	1.07	1.40	1.85	1.00
CW-L	8.82 ± 0.11	132.61 ± 6.65**	70.96 ± 4.42	219.52 ± 31.64**	1.27 ± 0.22	1.19	1.08	1.54	1.87	1.08
CW-H	8.87 ± 0.18	130.87 ± 6.29**	69.27 ± 8.40	290.06 ± 27.64**	1.48 ± 0.06	1.17	1.05	2.03	1.89	1.46

**p < 0.01 compared with the model group.

### Prolonged Diuretic Activity of Coconut Water on Rats

#### Effect of Prolonged Treatment With Coconut Water on Urine Volume in Rats

**FIGURE 1:**
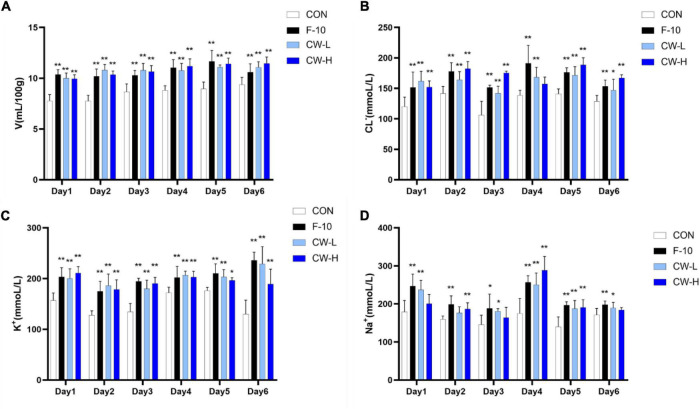
Prolonged diuretic effect of coconut water (CW) on urinary excretion volume **(A)**, urinary Cl^–^ **(B)**, urinary K^+^ **(C)** and urinary Na^+^ concentration **(D)** of rats. Values expressed as mean ± SD (*n* = 9), *p < 0.05 and **p < 0.01 compared to the control group.

To determine the prolonged diuretic activity of coconut water, the urinary excretion volume of rats in the CON, F-10, CW-L, and CW-H groups were determined for 7 consecutive days (Figure 1). The urine volume of F-10 and CW-L significantly increased within 6 days compared with the CON group (*P* < 0.01).

#### Effect of Prolonged Coconut Water Treatment on Urine Electrolyte Concentration in Rats

Compared with the CON group, the excretion levels of Na^+^, K^+^, and Cl^–^ significantly increased during 6 days after treatment with furosemide and CW. Meanwhile, urinary electrolyte excretion significantly increased in both CW-L and CW-H groups (Figure 1).

#### Effect of Prolonged Coconut Water Treatment on Renin–Angiotensin–Aldosterone System Pathways in Rats

**FIGURE 2:**
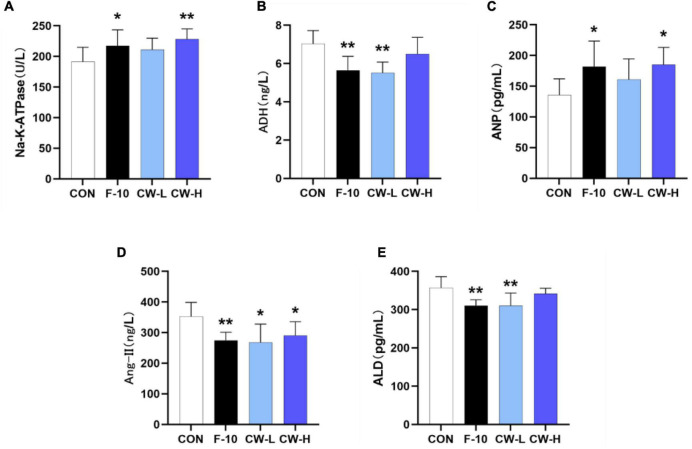
Effect of coconut water on Na^+^-K^+^-ATPase **(A)**, ADH **(B)**, ANP **(C)**, Ang II **(D)**, and ALD **(E)** in serum levels of rats. Values are expressed as mean ± SD (*n* = 9), *p < 0.05 and **p < 0.01 compared to the control group.

To determine the impact of prolonged CW intake on electrolyte excretions in rats, the serum Na^+^–K^+^–ATPase activity was determined (Figure 2A). As shown, F-10 and CW-H significantly increased the serum Na^+^–K^+^–ATPase activity, while CW-L did not significantly affect this enzyme activity in normal rats compared with the CON group.

To clarify the influence of CW treatment on the RAAS, the serum levels of ADH, ALD, Ang II, and ANP in rats were also determined (Figures 2B–E). It was observed that the ANP levels in serum were increased and ADH, ALD, and Ang-II were significantly decreased after treatment with F-10 (*P* < 0.05) compared with the CON group. Moreover, the CW markedly increased the ANP levels compared with the CON group, especially for the CW-H group (*P* < 0.05); CW-L significantly decreased the ADH and ALD levels (*P* < 0.01); and both CW-L and CW-H showed a significant reduction effect on Ang-II level (*P* < 0.05).

#### Effect of Coconut Water on Serum Aquaporins Levels

**FIGURE 3:**
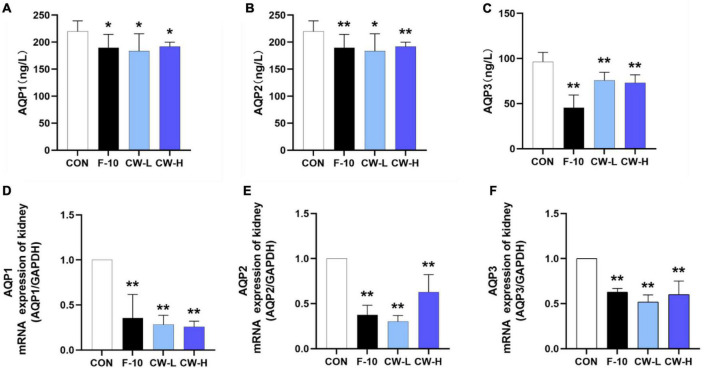
Effect of prolonged coconut water treatment on rat serum levels of aquaporin 1 (AQP1) **(A)**, AQP2 **(B)**, and AQP3 **(C)** and the mRNA expressions of AQP1, AQP2, and AQP3 in the kidney. The mRNA levels of AQP1, AQP2, and AQP3 were detected by RT-PCR. The mRNA levels of AQP1 **(D)**, AQP2 **(E)**, and AQP3 **(F)** were normalized to the model. Values are expressed as mean ± SD (*n* = 9), *p < 0.05 and **p < 0.01 compared to the control group.

To explore the regulatory role of CW on AQP1, AQP2, and AQP3 expressions, the AQP levels in rat serum were determined by ELISA kits. Compared with the CON group, the levels of AQP1, AQP2, and AQP3 in serum were significantly down-regulated after treatment with CW (*P* < 0.05, Figures 3A–C).

#### Coconut Water Suppressed AQP1, AQP2, and AQP3 mRNA Expressions

Meanwhile, the effects of CW on the mRNA expressions of AQP1, AQP2, and AQP3 in the kidney were also determined. Compared with CON group, treatment with F-10, CW-L, and CW-H suppressed AQP1, AQP2, and AQP3 mRNA expressions in rats (*P* < 0.01, Figures 3D–F).

#### Coconut Water Suppressed AQP1, AQP2, and AQP3 Protein Expressions

**FIGURE 4:**
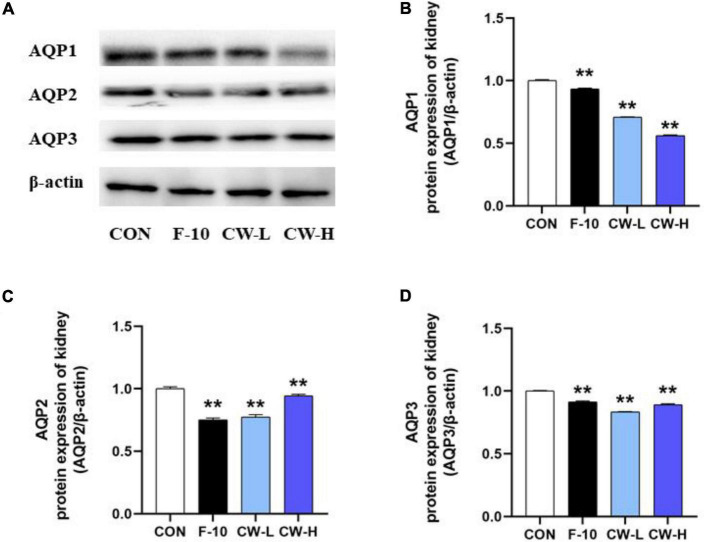
The levels of AQP1, AQP2, and AQP3 protein expression from different groups were detected by Western blot assay, and representative bands are shown in **(A)**. The levels of AQP1 **(B)**, AQP2 **(C)**, and AQP3 **(D)** were normalized to the model group. Values expressed as mean ± SD (*n* = 3), **p < 0.01 compared to the control group.

The AQP1, AQP2, and AQP3 protein expressions were evaluated after treatment with CW in rats (Figure 4). Obviously, CW administration could effectively down-regulate the related protein expressions, including AQP1, AQP2, and AQP3 in rats, which was in accordance with the levels in serum and mRNA expressions for AQP1, AQP2, and AQP3 (Figure 3). These results indicated that CW exerted significant diuretic effects by suppressing AQP1, AQP2, and AQP3 expressions in the kidney and serum.

## Discussion

Coconut water is a generally accepted tropical beverage with a pleasant flavor and a variety of beneficial nutrients including free amino acids and phytochemicals like flavonoids. Coconut water is reported to possess various pharmacological activities in health and medicinal applications (11, 12). For example, intravenous coconut water was effective in the treatment of hypertension, probably due to its potential diuretic effect. However, the underlying mechanisms remain unclear and restrict its utilization as a natural available diuretic to replace the synthetic furosemide, which is clinically and commonly used, frequently causing side effects. In the current work, the diuretic ability of coconut water and the mechanism were investigated.

As for the most intuitive phenomenon of urine excretion volume, the coconut water effectively increased the urine volume under the acute and/or prolonged treatment when compared with the control group. The increase in urine excretion was even more significant than the furosemide treatment, especially for the CW-L group. The diuretic activity could be reflected by the diuretic index. Briefly, the diuretic activity is considered good if the diuretic index is larger than 1.50, moderate if the value is between 1.00 and 1.50, mild if the value ranges from 0.72 to 1.00, and none with the value less than 0.72 (24). In the present study, after acute treatment for 24 h, the diuretic index for CW-L and CW-H group was 1.87 and 1.31, respectively. Thus, CW-L showed good diuretic potential while CW-H showed moderate diuretic potential. The diuretic activity value of CW-L was 1.19, which exhibited better diuretic activity compared to CW-H.

Na^+^, K^+^, Cl^–^, and Ca^2+^ are major electrolytes that exist in extracellular and intracellular fluids and contribute to maintaining the body’s fluid homeostasis. The Na^+^/K^+^ ratio was generally regarded as an index for natriuretic activity (25),and it implied satisfactory natriuresis, favorable natriuresis, and favorable K^+^ sparing activity, respectively, if this value larger than 1, 2, and 10 (26). In the current study, after administration of furosemide and coconut water for 24 h, the Na^+^/K^+^ ratio of F-10, CW-L, and CW-H groups was 1.85, 1.87, and 1.89, respectively, which indicated that coconut water could significantly increase this ratio, namely boost the Na^+^ excretion. Thus, it was supposed that the coconut water exhibited satisfactory natriuresis activity as furosemide, which could decrease the ability of renal concentration by inhibiting NaCl absorption in the thick ascending limb and enhance urine excretion carrying Na^+^ and Cl^–^ (27). The diuretic mechanism of the CAIs effect could be predicted by the urinary Cl^–^/Na^+^+K^+^ ratio (28). If the ratio lies between 0.8 and 1.0, it is not involved in the CAIs effect. However, if it is less than 0.8, it is considered to exhibit strong CAI activity (29). In the present study, this value was larger than 1, thus indicating that CAI activity was not involved in the diuretic mechanism of coconut water.

In addition, the acute treatment of CW significantly increased urinary Na^+^ and Cl^–^, while the K^+^ hardly changed, which implied the enhanced excretion of NaCl by CW and retention of K^+^. After prolonged CW treatment, the ratio of Na^+/^K^+^ of the urinary electrolyte was consistent with the acute treatment, which indicated that long-term consumption of coconut water results in an excellent diuretic effect with little urinary electrolyte fluctuations. Diuretic agents were widely used to regulate these disorders by inhibiting water and electrolytes reabsorption into the bloodstream across tubular epithelial cells (30). Therefore, the coconut water turned out to be more advantageous than diuretic drugs, which possibly cause electrolyte abnormality, especially for the authentic CW, which has similar osmotic pressure to rat blood (3).

The RAAS exerts a profound influence on body fluid regulation and effectively controls fluid homeostasis and maintains electrolyte balance. ADH, Ang II, and ALD are three main hormones released from RAAS, which could decrease the GFR and increase the Na^+^ reabsorption at the tubular (31, 32). ANP, a hormone released by the mammalian atria, has been reported to increase diuresis and Na^+^ electrolyte excretion, as well as suppress the activation of RAAS and block the release and/or actions of ALD and Ang II, and ALD promotes the reabsorption of Na^+^ and water in the kidney and is regulated by renin and Ang. Moreover, Ang II could also promote the reabsorption of Na^+^ reabsorption and the release of ADH (8, 32, 33). Thus, the urine excretion could be increased by suppressing RAAS and enhancing ANP levels. In the present study, CW observably decreased ADH, ALD, and Ang II levels, while it significantly increased the ANP level compared to the CON group, which eventually suppressed RAAS activation. In some previous studies, the ethanol extract of *Lagopsis supine* was also observed to exert a diuretic effect via a similar mechanism (9, 33).

It has been reported that AQP1, AQP2, and AQP3 emerge as three intrinsic membrane proteins in aquaporins that are associated with water transport, as well as primary urinary reabsorption (8). Thus, a slight change in AQPs could remarkably affect changes in urine volume attributing to more than 99% of the water in urine being reabsorbed (8, 34, 35). In the present study, CW remarkably decreased serum AQP1, AQP2, and AQP3 excretions and suppressed the mRNA and protein expressions of AQP1, AQP2, and AQP3 compared with the CON group in rats. This indicated that CW exhibited diuretic activity by regulating AQP1, AQP2, and AQP3 expressions in rats, which was in accordance with the previous studies (8, 9). These studies observed that flavonoids and/or phenylpropanoids from *Lagopsis supine*, *Bauhinia forficata* Link Leaves, and *Clerodendrum myricoides* serve as the key compounds that exhibit diuretic effects via the above mechanisms. Much evidence relating to the diuretic effects exerted by phytochemicals has been previously reported (36). In the present study, the CW phytochemicals analysis by LC-MS/MS indicated that flavonoids including apigetrin, baicalin, myricetin, catechin, L-epicatechin, procyanidin, and chlorogenic acid were abundant (Table 2). Moreover, amino acid intake can also be used to treat kidney disease or protect the kidneys based on the effective diuretic effect (37, 38). Thus, we speculated that the beneficial nutrients of CW, such as flavonoids, phenolic acids, and amino acids, serve as critical factors for its diuretic activity. However, further studies are also required to verify the presumptive diuretic activity of polyphenols like chlorogenic acid and abundant amino acids like phenylalanine.

## Conclusion

Coconut water could decrease serum ADH, ALD, and Ang II levels, while it significantly increases serum ANP level and down-regulates the mRNA expressions and protein levels of AQP1, AQP2, and AQP3. In a word, CW could exhibit acute and prolonged diuretic effects by suppressing the AQP and RAAS pathways in saline-loaded rats. This work provided the basic data for CW as an alternative diuretic agent to treat kidney diseases and replace traditional medicine. It is necessary for further studies to illustrate the active substances responsible for the diuretic activity of coconut water.

## Data Availability Statement

The raw data supporting the conclusions of this article will be made available by the authors, without undue reservation.

## Ethics Statement

The animal study was reviewed and approved by the Ethical Committee of Experimental Animal Care of Hainan University (HNUAUCC-2021-00108).

## Author Contributions

JW and MZ: methodology, software, validation, formal analysis, data curation, and writing – original draft preparation. KM: project administration. YP: Writing – review and editing. CL: investigation. GX and WZ: conceptualization, visualization, supervision, funding acquisition, and resources. All authors have read and agreed to the published version of the manuscript.

## Conflict of Interest

The authors declare that the research was conducted in the absence of any commercial or financial relationships that could be construed as a potential conflict of interest.

## Publisher’s Note

All claims expressed in this article are solely those of the authors and do not necessarily represent those of their affiliated organizations, or those of the publisher, the editors and the reviewers. Any product that may be evaluated in this article, or claim that may be made by its manufacturer, is not guaranteed or endorsed by the publisher.

## Supplementary Material

Supplementary Figure 1 Physicochemical property of coconut (*Cocos nucifera* L.cv.Wenye.No. 4) water.

Supplementary Figure 2 Effects of CW on the body weight **(A)**, food consumption **(B)** and water intake **(C)** of rats. Values expressed as mean ± SD (*n* = 9).

Supplementary Figure 3 The representative bands of AQP1, AQP2, and AQP3 protein expression from different groups were detected by Western blot.
