# Supplementary material for: The Diuretic Effects of Coconut Water by Suppressing Aquaporin and Renin–Angiotensin–Aldosterone System in Saline-Loaded Rats
Source: Front Nutr. 2022 Jun 23;9:930506. doi: 10.3389/fnut.2022.930506 (PMC9262403; doi:10.3389/fnut.2022.930506)

The diuretic effects of coconut water by suppressing aquaporin and renin-angiotensin-aldosterone system in saline-loaded rats

Supplementary information

Table 1S. Amino acids compositions in coconut water.

Amino acid	Content (mg/L)
Asp	0.26
Thr	5.04
Ser	15.25
Glu	31.46
Gly	5.50
Ala	151.54
Val	5.63
Cys	0.58
Met	1.69
Leu	0.94
Tyr	0.19
Lys	6.41
His	14.60
Arg	37.19
Pro	63.49
AspNH2	22.90
GluNH2	224.99
Trp	0.52
Total	588.17

Fig.1S. Physicochemical property of coconut (*Cocos nucifera* L.cv.Wenye.No.4) water.

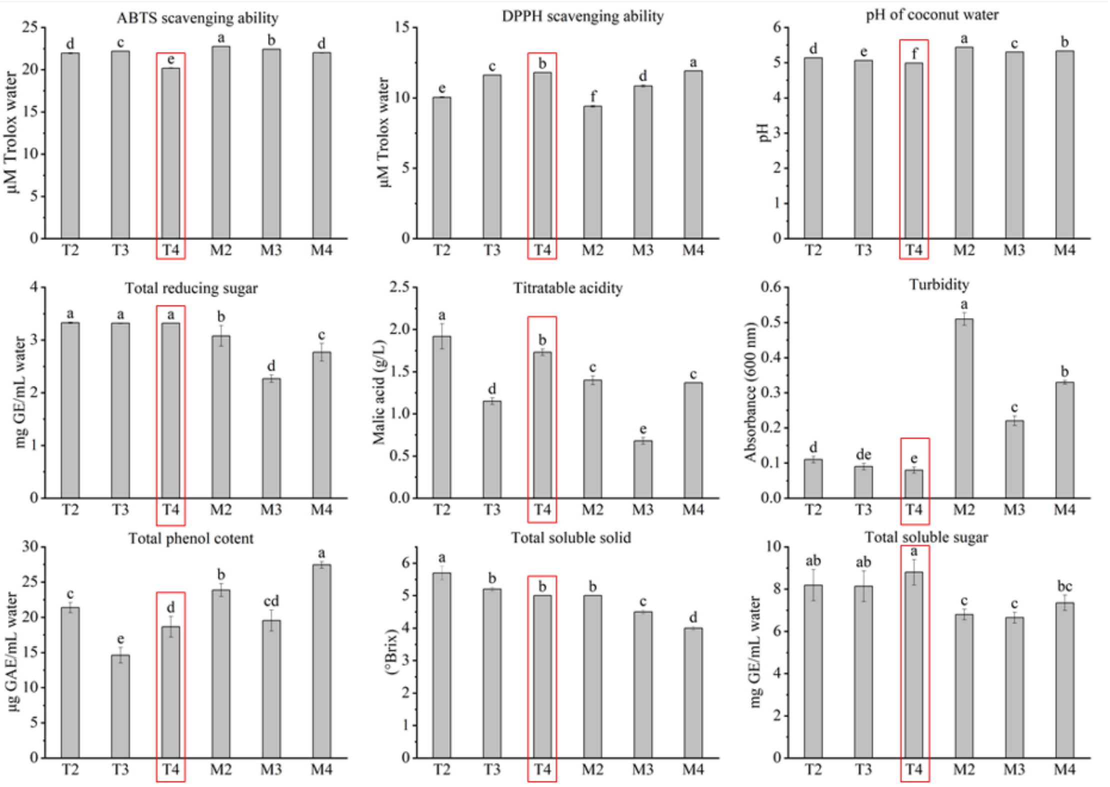

Fig.2S. Effects of CW on the body weight (A), food consumption (B) and water intake (C) of rats. Values expressed as mean ± SD (n = 9).

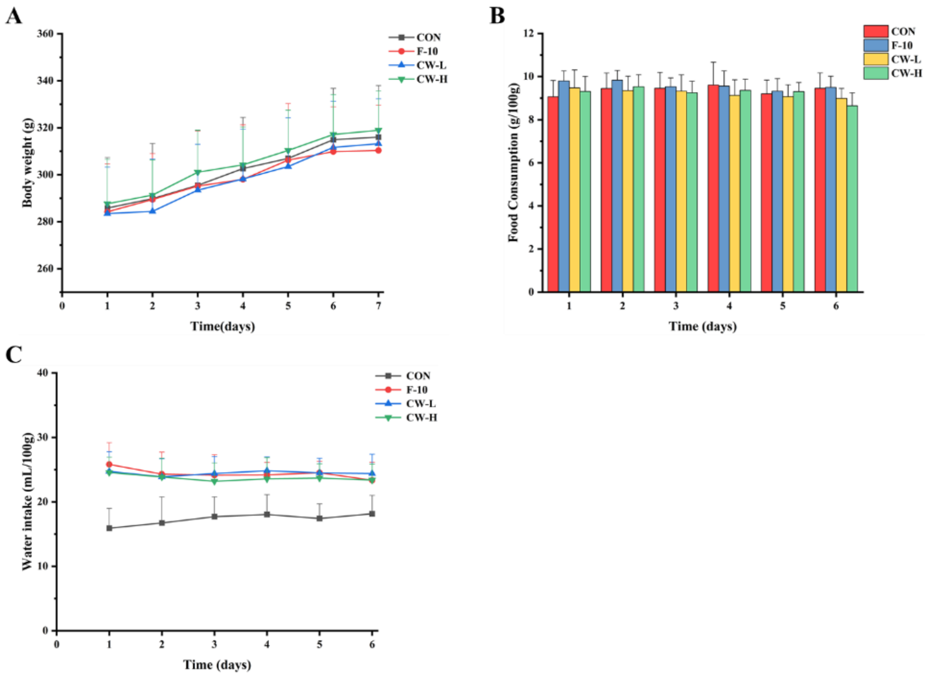

Fig.3S. The representative bands of AQP1, AQP2, and AQP3 protein expression from different groups were detected by Western blot.

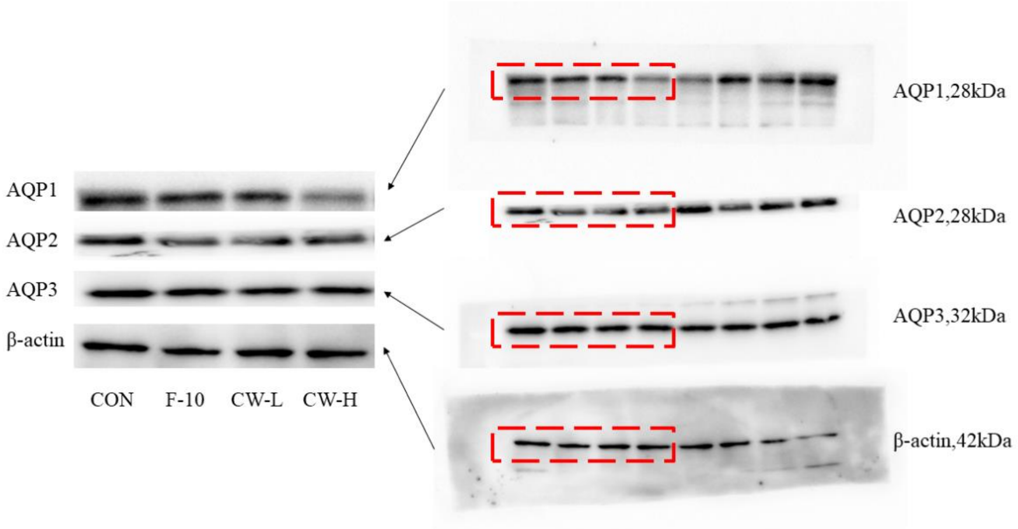

Supplement: Supplementary Figure 1 — Physicochemical property of coconut (Cocos nucifera L.cv.Wenye.No. 4) water. [file Data_Sheet_1.pdf]
